# Hepatoprotective Effects of Black Ginseng Extract and Ginsenoside Rh1 Against Alcohol-Induced Liver Injury: Mechanistic Insights from Network Pharmacology, In Vitro, and In Vivo Analysis

**DOI:** 10.3390/antiox15040461

**Published:** 2026-04-08

**Authors:** Hyeon Seon Na, Jeon Hwang-Bo, Woo-Cheol Shin, Jin-Kyu Jang, Bo-Ram Choi, Dae Young Lee

**Affiliations:** 1 BK21 FOUR KNU Creative BioResearch Group, School of Life Sciences, Kyungpook National University, Daegu 41566, Republic of Korea; hs1295@knu.ac.kr (H.S.N.); suc0094@knu.ac.kr (W.-C.S.); jkjang80@knu.ac.kr (J.-K.J.); 2 Department of Genetic Engineering, Graduate School of Biotechnology, Kyung Hee University, Yongin 17104, Republic of Korea; hbj3286@khu.ac.kr; 3 Department of Herbal Crop Research, National Institute of Horticultural and Herbal Science, Rural Development Administration, Eumseong 27709, Republic of Korea; bmcbr@korea.kr

**Keywords:** black ginseng extract, alcohol-induced liver damage (AILD), hepatoprotective effect, ginsenoside Rh1, LC–MS analysis, network pharmacology

## Abstract

Alcohol-induced liver damage (AILD), characterized by oxidative stress and inflammation, is a major health concern. While black ginseng extract (BGE) exhibits diverse pharmacological activities, its protective effects against AILD and underlying molecular mechanisms remain unclear. This study evaluated the protective effects of BGE against AILD using in vivo, in vitro, and in silico models. In mice, daily oral administration of 25% ethanol (5 g/kg) for 2 weeks induced liver injury. BGE (100–500 mg/kg) significantly reduced serum alanine aminotransferase (AST) and aspartate aminotransferase (ALT)levels while increasing catalase (CAT) and superoxide dismutase (SOD) activities. In ethanol-treated HepG2 cells, BGE inhibited nitric oxide (NO) production and suppressed cyclooxygenase-2 (COX-2), inducible nitric oxide synthase (iNOS), tumor necrosis factor-alpha (TNF-α), and interleukin-6 (IL-6) expression while increasing heme oxygenase-1 (HO-1)expression. Ginsenoside Rh1, quantified at 4.7 mg/g via quadrupole linear ion trap tandem mass spectrometry coupled with UPLC (UPLC-Q-TRAP-MS/MS), was identified as a key bioactive compound. Network pharmacology and molecular docking analyses revealed key inflammatory signaling pathways and core hub genes associated with ginsenoside Rh1. Integrated analyses suggest that ginsenoside Rh1 contributes to the multi-target effects of BGE by modulating inflammatory signaling pathways. Collectively, BGE is a potential therapeutic candidate for the prevention and treatment of AILD, with ginsenoside Rh1 serving as a key bioactive constituent and quality control marker.

## 1. Introduction

AILD is a major public health concern, representing a common form of chronic alcohol-induced liver disease that progresses from simple steatosis to alcoholic hepatitis, hepatic fibrosis and cirrhosis, and ultimately hepatocellular carcinoma [1]. Alcohol is primarily absorbed in the stomach and small intestine, transported to the liver, and metabolized. Hepatic alcohol metabolism occurs mainly through oxidative pathways that are mediated by three enzyme systems: alcohol dehydrogenase, cytochrome P450 2E1 (CYP2E1), and CAT [2,3]. This process induces CYP2E1, aldehyde oxidase, and other enzymes, leading to oxidative stress via the increased generation of reactive oxygen species (ROS) in vivo [4]. Oxidative stress triggers immune or inflammatory responses in several liver cell types, causing liver damage and disrupting the antioxidant defense balance, thereby contributing to various diseases [5,6]. To eliminate ROS, antioxidant enzymes such as CAT, glutathione peroxidase, SOD, glutathione reductase, and non-enzymatic antioxidants such as glutathione work in the liver cells. These antioxidants protect against free radical-induced oxidative damage. Similar to oxidative stress, inflammation contributes to the entire spectrum of liver disease from early to advanced stages [7]. NO is involved in numerous toxic oxidative reactions [8]. Alcohol consumption significantly elevates NO levels due to the upregulation of iNOS [9]. Therefore, increased NO generation serves as an early indicator of ethanol (EtOH)-induced liver injury, and inhibiting NO release is a potential method for controlling inflammation [10]. The induced expression of COX-2 contributes to inflammation and exacerbates AILD [8]. Long-term alcohol consumption activates innate immunity, leading to the production of proinflammatory cytokines, such as TNF-α, and the subsequent induction of hepatocellular damage [9,11,12,13]. Alongside this proinflammatory response, innate immunity activation in AILD upregulates hepatoprotective cytokines (e.g., IL-6) [14,15]. HO-1, a major antioxidant enzyme, plays a critical role against EtOH-induced oxidative stress in hepatocytes [16]. HO-1 induction inhibits key hepatic inflammatory mediators, including iNOS and COX-2, by modulating the janus kinase 2 (JAK2)/ signal transducer and activator of transcription 1 (STAT1) signaling [17,18]. Recent studies show that HO-1 upregulation increases carbon monoxide, a key downstream mediator, to prevent EtOH-induced hepatic inflammation [19]. This carbon monoxide acts as a significant regulator of signaling pathways that regulate liver inflammatory responses, such as mitogen-activated protein kinase (MAPK) and early growth response 1 (EGR1) [20].

Black ginseng (*Panax ginseng* C. A. Meyer), produced by repeatedly steaming and drying raw ginseng three to nine times, is formed via the Maillard reaction. The biological and pharmacological activities of steam-processed ginseng are greater than those of non-steamed ginseng. During the steaming and drying process, the percentage of primary constituents, including saponins, ginsenosides, proteins, and phenolics, is altered due to newly produced active compounds [21,22]. Black ginseng contains newly discovered ginsenosides and demonstrates more potent biological activities than those of white and red ginseng [23]. Previous studies have reported that the processed ginsenosides of black ginseng exhibit anticancer, antidiabetic, anti-inflammation, and neuroprotective activities [24,25,26,27]. However, the protective effect of BGE and its representative ginsenoside against AILD remains unexplored. Therefore, this study aims to comprehensively evaluate the hepatoprotective effects of BGE and major ginsenoside on EtOH-induced liver damage by integrating in vitro and in vivo experiments using HepG2 cells and an EtOH-induced liver injury mouse model, as well as in silico computer simulation analysis.

## 2. Materials and Methods

### 2.1. Chemicals and Reagents

Tissue culture reagents, including Roswell Park Memorial Institute 1640 (RPMI-1640) and fetal bovine serum (FBS), were purchased from Gibco BRL Co. (Grand Island, NY, USA). All other chemicals were obtained from Sigma-Aldrich Chemical Co. (St. Louis, MO, USA). The following primary antibodies were used: anti-iNOS (Abcam, Waltham, MA, USA, Cat no. ab153231, Lot no. GR3205303-2), anti-COX-2 (Santa Cruz, Dallas, TX, USA, Cat no. sc-376861, Lot no. A0819), anti-IL-6 (Santa Cruz, Dallas, TX, USA Cat no. sc-57315, Lot no. I0816), anti-TNF-α (Santa Cruz, Dallas, TX, USA, Cat no. sc-52746, Lot no. C0119), and anti-HO-1 (Abcam, Waltham, MA, USA, Cat no. ab13243). Peroxidase-conjugated anti-rabbit (St. Louis, MO, USA, Cat no. A0545) and anti-mouse (St. Louis, MO, USA, Cat no. A0168) secondary antibodies were obtained from Sigma-Aldrich. The distilled water and acetonitrile used for analysis were HPLC grade (Fisher Scientific Korea Ltd., Seoul, Republic of Korea), and the formic acid used in the analysis solvent was mass-spectrometry grade (Honeywell Fluka^TM^, Fisher Scientific, Waltham, MA, USA) with 99.0% purity. All other reagents and solvents were of first-grade or higher quality.

### 2.2. Preparation of Black Ginseng Extract (BGE)

Black ginseng was produced by steaming dried ginseng roots at 95 ± 2 °C, followed by drying at 40 °C for 6–8 h; this process was repeated four times, as described previously [28]. BGE powder was obtained from Daedong Korea Ginseng Co., Ltd. (Daejeon, Republic of Korea) and prepared by spray-drying extracts of dried black ginseng. The dried black ginseng was sequentially extracted at 85 °C for 8 h with 60%, 40%, and 30% (*v*/*v*) ethanol, followed by distilled water, with the residue from each step used for the next extraction. The voucher specimen (RDA19-01) was deposited in the herbarium of the Department of Herbal Crop Research, National Institute of Horticultural and Herbal Science, Rural Development Administration, Eumseong, Republic of Korea.

### 2.3. Animals and Study Design

The animal facility and study protocol (KHUASP-18-006, approval date: 16 March 2018) were approved by the Kyung Hee University Institutional Animal Care and Use Committee. Animal care and experimental procedures were conducted according to the Kyung Hee University guidelines for the care and use of laboratory animals. Female BALB/c mice (5 weeks old, *n* = 35) were purchased from Raon Bio Inc. (Yongin, Republic of Korea). All mice were clinically healthy and had not undergone any previous experimental procedures prior to inclusion in this study. The genotype was wild type. Mice received water and food ad libitum while quarantined in a controlled environment with a 12 h light/dark photoperiod.

Mice were randomly assigned to five experimental groups: control (*n* = 7), EtOH-only (*n* = 7), EtOH + BGE 100 mg/kg (*n* = 7), EtOH + BGE 300 mg/kg (*n* = 7), and EtOH + BGE 500 mg/kg (*n* = 7). Each group was treated with 25% EtOH (*w*/*v*, 5 g/kg bw/day; Merck Milipore, Billerica, CA, USA) via oral gavage for 14 days. Mice were treated with BGE (0, 100, 300, and 500 mg/kg) for 17 consecutive days, beginning 3 days before EtOH administration. All mice were monitored daily for 17 days. At the end of the experiment, the mice were euthanized, blood and tissues were collected, and serum was obtained through centrifugation (2500× *g*, 15 min). The liver, spleen, and kidneys were carefully excised, and tissues were weighed after removing as much blood as possible with a paper towel.

Mice were randomly assigned to five experimental groups in a balanced manner to ensure that the average body weight was equivalent across all groups. To avoid selection bias, groups were established prior to the commencement of any treatments. The histological assessments and biochemical analyses were conducted using coded samples to minimize potential observer bias. The animal or cage location was not considered as a controlled variable. The inclusion criteria were predefined as clinically healthy animals within a specific weight range that successfully completed the protocol. Although exclusion criteria—such as treatment-related illness, technical errors, or anesthesia complications—were established, no animals or tissues met these criteria. Consequently, all were included in the final analysis. For statistical analyses, the exact sample size was *n* = 7 per experimental group, which was determined based on our previous experience and preliminary studies to provide sufficient statistical power for detecting significant differences in primary outcomes. No expected or unexpected adverse events occurred, and the predefined humane endpoints were not triggered, as no animals exhibited clinical signs requiring early euthanasia. The primary outcome measures in the animal studies were antioxidant enzyme (CAT, SOD) activity and the biochemical markers of hepatotoxicity (ALT, AST, LDH) measured in serum and liver tissue.

### 2.4. Enzyme-Linked Immunosorbent Assay

Serum alanine aminotransferase (ALT; BioVision Inc., Milpitas, CA, USA, Cat No. K752-100, Lot 4A10K07520) and aspartate aminotransferase (AST; BioVision Inc., Milpitas, CA, USA, Cat No. K753-100, Lot 4C28K07530) activities were measured using colorimetric assay kits, following the instructions of the manufacturer. Hepatic CAT, SOD, and lactate dehydrogenase (LDH) activities were determined using CAT (Abcam, Waltham, MA, USA, Cat no. ab83464, Lot no. GR3212922-2) and SOD (Abcam, Waltham, MA, USA, Cat no. ab65354, Lot no. GR3190964-4) colorimetric assay kits and an LDH activity assay kit (Sigma-Aldrich, St. Louis, MO, USA, Cat no. MAK066), following the instructions of the manufacturer.

### 2.5. Histopathological Examination

The liver tissues were fixed in 4% paraformaldehyde solution and embedded in paraffin. Paraffin-embedded tissues were sectioned at 5 μm. The paraffin sections were stained with hematoxylin and eosin (H&E) to assess the histopathological changes. Sections were then mounted with mounting medium (Invitrogen, Carlsbad, CA, USA), cover-slipped, and examined under a light microscope (Nikon, Tokyo, Japan).

### 2.6. Cell Culture and Treatment

HepG2 human liver hepatocellular carcinoma cells were obtained from the Korean Cell Line Bank (KCLB, Seoul, Republic of Korea) and cultured in Dulbecco’s modified Eagle’s medium (HyClone, Logan, UT, USA) supplemented with 10% FBS (Gibco BRL Co.) at 37 °C in a 5% CO_2_ humidified incubator.

For the in vitro experiments, HepG2 cells were pretreated with various concentrations of BGE (dissolved in PBS) in serum-free medium for 1 h. Subsequently, the cells were exposed to 0.5 M EtOH for 24 h in the presence of BGE. The control cells were treated with an equal volume of PBS (vehicle control).

### 2.7. Cell Viability Assay

The cell viability of BGE was assessed using the MTT colorimetric assay. HepG2 cells (5 × 10^4^ cells/mL) were treated with varying concentrations of BGE and HDE (as a positive control with known hepatoprotective effects) for 24 h. Twenty microliters of MTT solution (5 mg/mL in PBS) were then added to each well, and the plate was incubated at 37 °C for 1 h. The medium was removed, and 100 μL of DMSO (Duchefa, Haarlem, The Netherlands) were added to each well. Formazan formation was quantified by measuring absorbance at 540 nm, using an EL800 Universal Microplate Reader (Bio-Tek Instruments Inc., Winooski, VT, USA).

$$\mathrm{Cell}\mathrm{viability}\left(\%\right)=\left[\frac{{{OD}_{BGE-treated}-OD}_{blank}}{OD_{control}-{OD}_{blank}}\;\right]\times 100$$

### 2.8. Nitric Oxide Assay

The nitrite concentration from HepG2 cells was determined using the Griess reagent system (Promega, Madison, WI, USA), following the instructions of the manufacturer. Absorbance at 550 nm was measured using an EL800 Universal Microplate Reader (Bio-Tek Instruments Inc.).

### 2.9. Reverse Transcription–Polymerase Chain Reaction Analysis

The total RNA was isolated from HepG2 cells using TRIzol Reagent (Invitrogen, Carlsbad, CA, USA), following the manufacturer-provided protocol. One microgram of the total RNA was reverse-transcribed into cDNA using an AccuPowerCycleScript RT Premix (Bioneer Inc., Daejeon, Republic of Korea). A PCR was performed using DreamTaq DNA polymerase (Thermo Fisher Scientific Inc., Waltham, MA, USA) with 2-μL cDNA and gene-specific primers. The primer sequences were as follows: iNOS, 5′-GCACATTTGGCAATGGAGAC-3′ (forward) and 5′-TGGACTTCTCACTCTGCAGA-3′ (reverse); COX-2, 5′-AGTCCCTGAGCATCTACGGT-3′ (forward) and 5′-TTCTGTACTGCGGGTGGAAC-3′ (reverse); HO-1, 5′-ATGGGTCCTTACACTCAGCT-3′ (forward) and 5′-AAACTCAGGGCTTTTGGAGG-3′ (reverse); TNF-α, 5′-CAAGACCACCACTTCGAAAC-3′ (forward) and 5′-GCAATGAGTGACAGTTGGTC-3′ (reverse); IL-6, 5′-CAGAGCTGTGCAGATGAGTA-3′ (forward) and 5′-ACTGCATAGCCACTTTCCAT-3′ (reverse); and β-actin, 5′-ATGTTTGAGACCTTCAACAC-3′ (forward) and 5′-CACGTCACACTTCATGATGG-3′ (reverse). PCR products were separated on 1% agarose gels that were electrophoresed, then visualized with ethidium bromide. The band intensities were determined using ImageJ software ver. 1.51k) (NIH, Rockville Pike, MD, USA).

### 2.10. Protein Extraction and Western Blot Analysis

Cells were washed with PBS and lysed in RIPA buffer (Pierce, Rockford, IL, USA) supplemented with a protease inhibitor cocktail (Sigma-Aldrich). Equal amounts of total protein (50 μg) were separated using 8% and 10% SDS-PAGE and transferred onto PVDF membranes (PALL, Port Washington, NY, USA). Membranes were blocked with 3% skim milk in TBS containing 0.1% Tween 20 for 1 h, and then incubated overnight at 4 °C with the primary antibodies (anti-iNOS, anti-COX-2, anti-IL-6, anti-HO-1, and anti-β-actin; Santa Cruz Biotech. Inc., Dallas, TX, USA), diluted 1:2000 in blocking solution. The membranes were also incubated with peroxidase-conjugated secondary antibodies (anti-mouse and anti-rabbit antibodies; Sigma-Aldrich) diluted 1:5000 in blocking solution. Protein bands were detected using SuperSignal West Pico and/or Femto PLUS chemiluminescent substrates (Thermo Fisher Scientific Inc.).

### 2.11. Method for the Extraction and Isolation of Ginsenoside Rh1

Ginsenoside Rh1 (Rh1) was isolated from BGE using successive in-house chromatographic techniques. The isolated compound appeared as a white powder with a purity exceeding 99%. Structural identification was carried out using electrospray ionization coupled with quadrupole time-of-flight mass spectrometry (ESI–Q–TOF/MS), showing an ion signal at *m*/*z* 683.478, corresponding to [M+HCOO]^−^. The structure was further verified by comparison with previously reported NMR data [29]. For extraction, 100 mg of the powdered BGE was mixed with 1 mL of 70% methanol in a 2 mL microcentrifuge tube. The sample mixture was sonicated in an ultrasonic bath (Bransonic 3510R-DTH, Branson Ultrasonics Co., Banbury, Charlotte, NC, USA) at 50 °C for 30 min. After centrifugation at 15,000 rpm for 5 min using a centrifuge (HITACHI Koki Co., Ltd., Tokyo, Japan), 1 mL of the supernatant was collected, and the extraction procedure was repeated once under the same conditions. The combined supernatants (2 mL) were filtered through a 0.2 μm, 13 mm syringe filter (Chromdisc Co., Daegu, Republic of Korea) before analysis. For instrumental analysis, Rh1 stock solution (1 mg/mL) was prepared, using methanol as the solvent.

### 2.12. UPLC–MS-Based Ginsenoside Profiling and Targeted Quantification of Rh1 in BGE

Ginsenoside profiling was performed using Q-TOF/MS operated in negative ion mode and coupled to a UPLC system, following the previously reported chromatographic and mass spectrometric conditions and column specifications [30]. Using MassLynx software (v4.2), MS data were acquired, and the resulting data were processed with UNIFI software (version 3.8.0.23; Waters Corp., Milford, MA, USA). For quantitative analysis, Rh1 and the BGE were diluted to concentration ranges of 0.156 to 2.5 ppm and 5000 ppm, respectively, and analyzed instrumentally. UPLC-Q-TRAP-MS/MS quantification was conducted using a 3200 QTRAP mass spectrometer (AB SCIEX, Framingham, MA, USA) fitted with a Turbo V™ ion source and Turbo Ion Spray probe and operated in negative electrospray ionization mode (−ESI). The chromatographic conditions were identical to those used in the UPLC–QTOF/MS profiling analysis described above. Quantification was conducted in multiple reaction monitoring (MRM) mode by selecting precursor ions, applying collision energy, and monitoring corresponding product ions. Table S1A summarizes key instrumental parameters. Operating conditions were defined with a curtain gas flow rate of 20 L/h and an ion source gas flow rate of 50 L/h, while the collision gas was maintained at medium. The ion spray voltage and ion source temperature were set to −4.5 kV and 450 °C, respectively. Instrument operation and data acquisition were carried out with BioAnalyst™ software (version 1.4.2, AB SCIEX).

### 2.13. Network Pharmacology

#### 2.13.1. Prediction of Compound-Associated Targets

The potential protein targets of the identified compounds were predicted using SwissTargetPrediction (http://www.swisstargetprediction.ch/, accessed on 28 October 2025), PubChem (https://pubchem.ncbi.nlm.nih.gov/, accessed on 28 October 2025), and the SEA Search Server (https://sea.bkslab.org/, accessed on 28 October 2025). All predicted targets were integrated, and duplicates were removed to generate a consolidated list of compound-associated targets.

#### 2.13.2. Identification of Alcoholic Liver Disease-Associated Targets

The GeneCards database (http://www.genecards.org, accessed on 28 October 2025) was searched using the keyword “Alcoholic liver disease” to identify genes associated with alcoholic liver disease. Overlapping targets between the predicted compound-associated targets and alcoholic liver disease-related genes were identified using a Venn diagram generated with Venny 2.1.0.

#### 2.13.3. Construction of the Protein–Protein Interaction Network and Identification of Core Hub Genes

Using STRING (v12.0), a protein–protein interaction (PPI) network was generated for the common targets with the minimum interaction confidence score set at >0.7. The PPI network was visualized and analyzed in Cytoscape (v3.10.4). The common targets were screened using the CytoHubba (v0.1) plugin, based on four topological parameters—betweenness, bottleneck, stress, and radiality. The top 10 targets ranked according to each parameter were further integrated, and the core hub genes were identified using a Venn diagram generated with Venny 2.1.0.

#### 2.13.4. Functional Enrichment Analysis Based on GO and KEGG Pathways

Gene Ontology (GO) enrichment analysis was conducted using ShinyGO (v0.85.1) to explore the enriched biological process, molecular function, and cellular component categories among the common targets. In addition, enrichment analysis of pathways in the Kyoto Encyclopedia of Genes and Genomes (KEGG) database was performed to investigate signaling pathways related to these targets [31,32,33].

#### 2.13.5. Molecular Docking Analysis

The chemical structure of Rh1 was drawn using ChemDraw (v19.0), converted to a 3-dimensional format, and saved as an SDF file. Corresponding protein targets were retrieved from UniProt (https://www.uniprot.org/, accessed on 26 November 2025). For each core hub gene, the highest-resolution human protein structure was obtained from the Protein Data Bank (PDB; https://www.rcsb.org/, accessed on 26 November 2025). Before molecular docking, water molecules and co-crystallized ligands were removed from the protein structures using PyMOL (v3.1). Molecular docking analysis was performed using CB-DOCK2 (https://cadd.labshare.cn/cb-dock2/php/index.php, accessed on 26 November 2025) to predict the binding affinities between Rh1 and the core hub target genes. Docking interactions and binding modes were visualized with BIOVIA Discovery Studio Visualizer (v25.1.0.24284).

To validate the docking protocol, the co-crystallized ligand was removed from the protein structure and re-docked using the same docking parameters described above. The docking accuracy was evaluated by calculating the heavy-atom root-mean-square deviation (RMSD) between the re-docked ligand pose and the original crystallographic ligand conformation after the removal of hydrogen atoms, using PyMOL (v3.1).

### 2.14. Statistical Analysis

All data were derived from three independent biological replicates, each analyzed in duplicate technical measurements for in vitro experiments, and from seven animals per group in in vivo studies. Data are expressed as the mean ± standard deviation (S.D.). The Shapiro–Wilk test was applied to assess normality. As all variables followed a normal distribution, the differences between the two groups were analyzed using Student’s *t*-test, while comparisons among multiple groups were performed using one-way ANOVA followed by Tukey’s post hoc test. For the in vivo experiments, the statistical analyses were performed using GraphPad Prism (v8.0). Statistical significance was defined as # *p* < 0.05, ## *p* < 0.01, and ### *p* < 0.001 versus control, and * *p* < 0.05, ** *p* < 0.01, and *** *p* < 0.001 versus EtOH-fed mice.

For quantitative analysis of measured compounds, data are expressed as mean ± standard deviation (S.D.). Differences between groups were evaluated using Student’s *t*-test or one-way ANOVA, followed by Tukey’s multiple comparison test. Significance was defined as *p* < 0.05, *p* < 0.01, and *p* < 0.001.

## 3. Results

### 3.1. Effects of BGE on Body Weight, Organ Weight, and Histopathological Changes in Ethanol-Fed Mice

**Figure 1:**
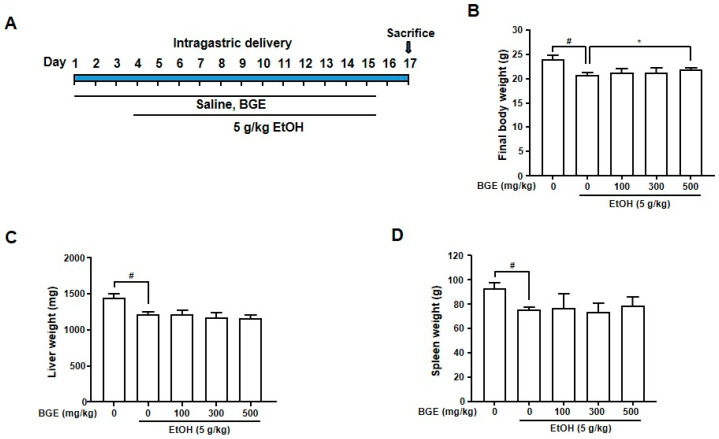
Effects of BGE on the final body and organ (liver and spleen) weights in EtOH-fed mice. (**A**) An experimental design used to evaluate the hepatoprotective effects of BGE against alcohol-induced liver damage. (**B**–**D**) At the end of the experimental period, mice were weighed and euthanized. The liver and spleen were carefully excised and weighed. Data are presented as mean ± SD (*n* = 7 mice per group). ^#^ *p* < 0.05 vs. control and * *p* < 0.05 vs. EtOH-fed mice.

The in vivo hepatoprotective effects of BGE were evaluated using an intragastric EtOH-feeding animal model. Hepatic dysfunction was induced through daily intragastric administration of 25% (*w*/*v*) EtOH (5 g/kg body weight) with or without BGE (0, 100, 300, or 500 mg/kg) for 14 days (Figure 1A). At the end of the experiment, body weight and the liver and spleen weights were recorded. Compared to controls, EtOH-fed mice showed reduced body weight, whereas BGE-treated groups exhibited a slight recovery (Figure 1B). Livers from the EtOH-fed group were slightly paler than those from the controls, although the liver weights did not differ significantly among groups. Spleen weight was reduced by EtOH feeding but was restored to near-control levels following BGE administration (Figure 1C,D).

**Figure 2:**
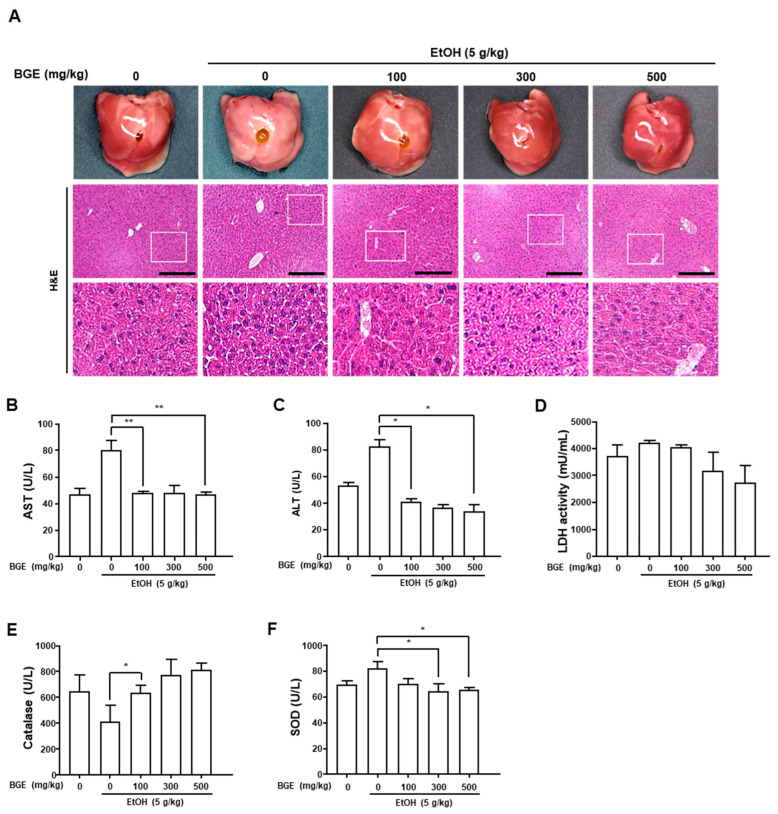
Effects of BGE on histopathological changes, hepatotoxicity markers, and oxidative stress in the livers of EtOH-fed mice. (**A**) Livers were excised and imaged using a digital camera. Liver sections were stained with hematoxylin and eosin, and images were captured at 100× and 400× magnifications (scale bar = 200 μm). (**B**–**D**) The serum levels of hepatotoxicity markers, including AST, ALT, and LDH, were quantified using colorimetric methods. (**E**,**F**) Activities of antioxidant enzymes, including CAT and SOD, were quantified in liver tissues using colorimetric assays. Data are presented as mean ± SD (*n* = 7 mice per group). * *p* < 0.05 and ** *p* < 0.001 vs. only EtOH-fed mice.

Histopathological changes in liver tissue were assessed through H&E staining. Under light microscopy, hepatocyte cytoplasm stained red, lipid droplets appeared as unstained vacuoles, and nuclei stained purple. Fat accumulation in liver tissue was observed only in the EtOH-fed group. In mice co-treated with EtOH and BGE, hepatic fat accumulation decreased in a dose-dependent manner compared to the EtOH-only group. These findings indicate the hepatoprotective effects of BGE against alcoholic steatosis (Figure 2A).

### 3.2. Effect of BGE on the Activities of Aspartate Aminotransferase, Alanine Aminotransferase, and Lactate Dehydrogenase in Ethanol-Fed Mice

In this experiment, serum AST, ALT, and LDH activities were analyzed as biochemical indicators of hepatotoxicity. Compared to the control group, the EtOH-fed group showed significantly elevated AST (53.2 ± 2.7 U/L vs. 82.5 ± 5.4 U/L) and ALT (47.0 ± 4.4 U/L vs. 80.5 ± 7.2 U/L) levels. These increases were dose-dependently attenuated through BGE administration (Figure 2B,C). LDH activity was also increased in the EtOH-fed group (4235.2 ± 102.5 mU/mL) compared to the control group, but was significantly reduced through BGE treatment at 300 and 500 mg/kg (3166.983 ± 690.8 and 2735.3 ± 639.5 mU/mL, respectively; Figure 2D). These findings indicate that BGE mitigates EtOH-induced elevations in AST, ALT, and LDH, demonstrating a hepatoprotective effect against alcoholic liver injury.

### 3.3. Effect of BGE on the Activities of Antioxidant Enzymes in Ethanol-Fed Mice

To evaluate the antioxidant effects of BGE on AILD, CAT and SOD activities were measured in the liver tissue. Compared to the control group, EtOH-fed mice showed reduced CAT (646.3 ± 126.3 U/L vs. 409.5 ± 126.5 U/L) and SOD (69.4 ± 3.8 U/L vs. 64.5 ± 1.1 U/L) activities. These enzyme activities were markedly increased in mice that were co-treated with BGE and EtOH (Figure 2E,F). The decrease in CAT and SOD activities in EtOH-fed mice is likely attributable to EtOH-induced free radical accumulation, whereas BGE appears to exert hepatoprotective effects against alcoholic oxidative stress by enhancing the antioxidant enzyme activity.

### 3.4. Cytotoxicity and Protective Effect of BGE in HepG2 Cells

**Figure 3:**
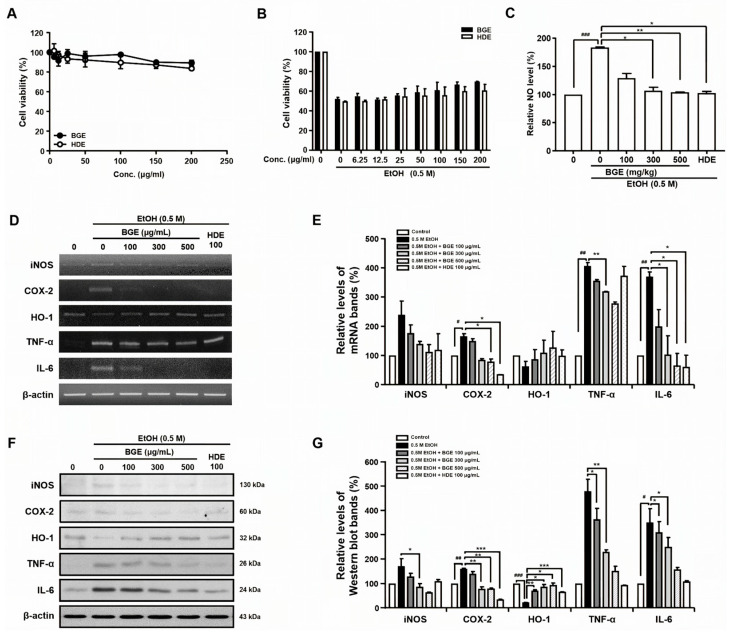
Effects of BGE on cytotoxicity, NO production, inflammatory mediators (iNOS, COX-2, TNF-α, and IL-6), and hepatoprotective enzyme (HO-1) in EtOH-treated HepG2 cells. (**A**) Cell viability of HepG2 cells treated with 0.5 M EtOH for 24 h following BGE pretreatment at various concentrations. (**B**) Cell viability of HepG2 cells treated with various concentrations of BGE for 24 h, measured using MTT assay. (**C**) NO production in HepG2 cells treated with 0.5 M EtOH for 24 h following BGE pretreatment at various concentrations, measured using a Griess reagent. (**D**) PCR of iNOS, COX-2, HO-1, TNF-α, IL-6, and β-actin, using specific primers. (**E**) Quantification of PCR products from three independent experiments (**D**) represented as a bar diagram. Transcript levels of iNOS, COX-2, HO-1, TNF-α, and IL-6 in the control were set to 100%. (**F**) Protein levels of iNOS, COX-2, HO-1, TNF-α, and IL-6 were determined using Western blot analysis with anti-iNOS, anti-COX-2, anti-HO-1, anti-TNF-α, and anti-IL-6. (**G**) Relative protein levels of iNOS, COX-2, HO-1, TNF-α, and IL-6 were quantified and presented as a bar diagram. Protein levels in control cells were set to 100%. Data are presented as mean ± SD from three independent biological experiments, each performed in duplicate (two technical replicates). ^#^ *p* < 0.05, ^##^ *p* < 0.01, ^###^ *p* < 0.001 vs. control, * *p* < 0.05, ** *p* < 0.01, and *** *p* < 0.001 vs. EtOH-only-treated HepG2 cells.

To evaluate the hepatoprotective effects of BGE in vitro, its cytotoxicity in HepG2 cells and its protective effect against EtOH-induced injury were assessed. The cell viability was measured relative to the untreated controls, and BGE showed no cytotoxicity at any tested concentration (0, 25, 50, 100, 200, 300, 400, and 500 μg/mL) (Figure 3A). Based on preliminary experiments, 0.5 M EtOH—reducing cell viability by 40–50% compared to the controls—was selected to induce HepG2 cell injury. With the control cell viability set at 100%, the EtOH treatment alone decreased viability by 53.1%. However, co-treatment with EtOH and BGE (300, 400, and 500 μg/mL) increased cell viability by 16.3%, 18.9%, and 35.5%, respectively, compared to EtOH treatment alone (Figure 3B). Notably, BGE-treated cells showed significantly higher viability than cells treated with the same concentration of the positive control, HDE, which is known for its hepatoprotective effects. These findings indicate that BGE protects hepatocytes from EtOH-induced injury.

### 3.5. Anti-Inflammatory Effects of BGE on Ethanol-Treated HepG2 Cells

Alcohol-induced oxidative stress triggers immune and inflammatory responses in liver cells, contributing to hepatic injury [5,6]. To assess the anti-inflammatory effects of BGE, inflammatory mediators (NO, iNOS, TNF-α, IL-6, and COX-2) were measured in EtOH-treated HepG2 cells. NO production was quantified using the Griess reagent system. The NO levels were significantly elevated by EtOH treatment and were reduced to 65.2%, 91.9%, and 95% by BGE at 100, 300, and 500 μg/mL, respectively (Figure 3C). The expression of inflammatory mediators was assessed using RT-PCR and Western blotting. Their mRNA and protein levels were significantly increased in EtOH-treated HepG2 cells, and attenuated through BGE treatment (Figure 3D–G). Additionally, the effects of BGE on the expression of HO-1, known to inhibit liver inflammatory mediators, including iNOS and COX-2, and prevent ethanol-induced inflammation through increased carbon monoxide, was confirmed by RT-PCR and Western blot. HO-1 expression in both mRNA and protein levels was reduced in EtOH-treated cells compared to control cells, but was significantly upregulated through BGE treatment (Figure 3D–G). These findings suggest that BGE may be beneficial in alleviating inflammatory AILD.

### 3.6. Protective Effects of Active Compound on Ethanol-Treated HepG2 Cells

**Figure 4:**
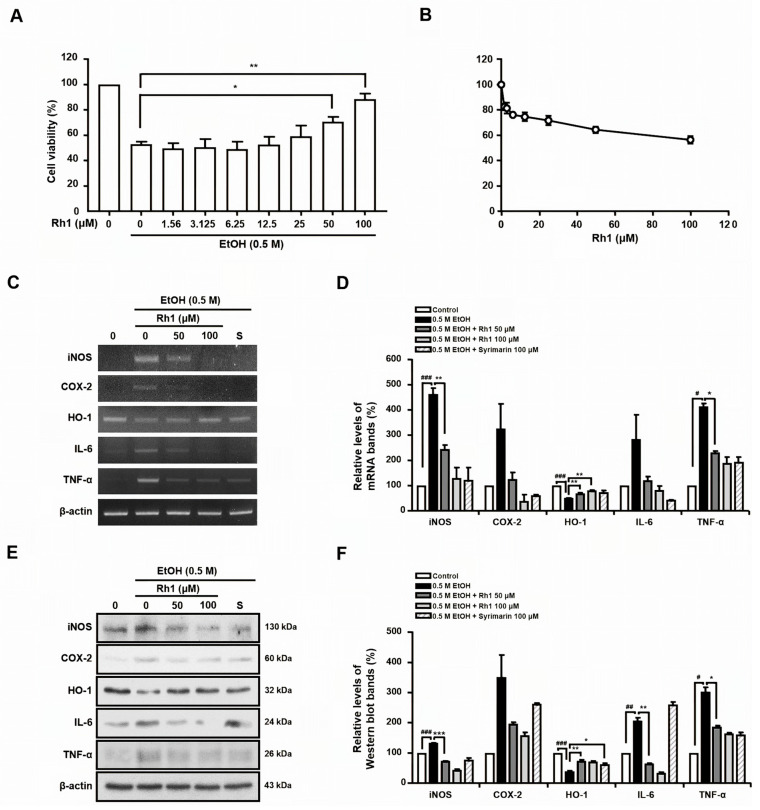
Effects of Rh1 on cytotoxicity, inflammatory mediators (iNOS, COX-2, TNF-α, and IL-6), and hepatoprotective enzyme (HO-1) in EtOH-treated HepG2 cells. (**A**) Cell viability of HepG2 cells treated with 0.5 M EtOH for 24 h following Rh1 pretreatment at various concentrations. (**B**) Cell viability of HepG2 cells treated with various concentrations of Rh1 for 24 h, measured using MTT assay. (**C**) PCR of iNOS, COX-2, TNF-α, IL-6, HO-1, and β-actin using specific primers. (**D**) Quantification analysis of PCR products, shown as a bar diagram. Transcript levels in the control were normalized to 100%. (**E**) Protein levels of iNOS, COX-2, HO-1, TNF-α, and IL-6 were determined using Western blot analysis. (**F**) Relative protein levels were quantified and are represented as a bar diagram. Protein levels in the control were set to 100%. Data are presented as a mean ± SD from three independent biological experiments, each performed in duplicate (two technical replicates). ^#^ *p* < 0.05, ^##^ *p* < 0.01, ^###^ *p* < 0.001 vs. control, * *p* < 0.05, ** *p* < 0.05, and *** *p* < 0.05 vs. EtOH-only-treated HepG2 cells. S: sylimarin 100 μM.

BGE profiling revealed Rh1 as an active compound. To evaluate its protective effect against AILD, the HepG2 cells were pretreated with Rh1 and then exposed to 0.5 M EtOH for 24 h. The EtOH treatment reduced cell viability by 47.3%, relative to controls, whereas the Rh1 pretreatment increased viability by 38.3% and 75.6% at 50 and 100 μM, respectively (Figure 4A,B).

Additionally, the effects of Rh1 on the expression of inflammatory mediators (iNOS, COX-2, IL-6, TNF-α, and HO-1) in EtOH-treated HepG2 cells were assessed using RT-PCR and Western blotting. Silymarin, a natural compound with known hepatoprotective activity, was used as a positive control. Inflammatory mediators’ expression was markedly increased in the mRNA and protein levels in EtOH-treated HepG2 cells, and these elevations were attenuated by Rh1 (Figure 4C–F). HO-1 expression, reduced by EtOH in the mRNA and protein levels, was significantly restored through Rh1 treatment (Figure 4C–F). These findings indicate that Rh1, the active compound of BGE, protects against inflammatory AILD.

### 3.7. Ginsenoside Profiling and Quantitative Determination of Rh1 in BGE

**Figure 5:**
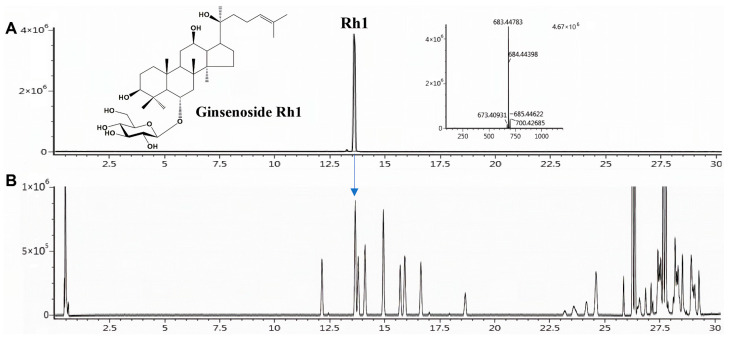
UPLC–Q-TOF/MS chromatograms of the (**A**) ginsenoside Rh1 and (**B**) black ginseng extract in negative ESI mode. The arrow indicates the Rh1 peak detected at 13.6 min in the black ginseng extract.

The ginsenoside composition of BGE was profiled, and targeted quantitative analysis of Rh1 was performed using the UPLC-Q-TOF/MS and UPLC-Q-TRAP-MS/MS system. In-house library [30] matching identified 24 ginsenosides in BGE, and under optimized conditions, Rh1 was detected at 13.6 min based on retention time and molecular mass comparison (Figure 5). Rh1 was selected as a marker analyte, whereas ginsenosides Rg3, Rg5, and Rk1, which are known markers of black ginseng [34], exhibited the highest relative abundances, followed by Rh1 (Table S2). For Rh1, the [M+COOH]^−^ ion was selected as the precursor ion and detected at *m*/*z* 683.44783 in the −ESI mode (Figure 5). Under optimized conditions, Rh1 was detected at a retention time of 13.42 min. The [M-H]^−^ ion (*m*/*z* 637.2) was selected as the precursor ion in the −ESI mode, and its MS/MS fragmentation patterns were analyzed (Table S1B). Quantification was conducted in the MRM mode, using optimal collision energy and the most sensitive product ion (*m*/*z* 160.9) (Table S1A). Calibration curves were constructed via linear regression using five concentrations of the standard. The coefficient of determination (r^2^) for Rh1 was 0.9998, indicating excellent linearity across the tested concentration range. The Rh1 content in the BGE was 4.73 mg/g (Table S1C).

### 3.8. Network Pharmacology Analysis

#### 3.8.1. Identification of Core Hub Genes from the Protein–Protein Interaction Network

**Figure 6:**
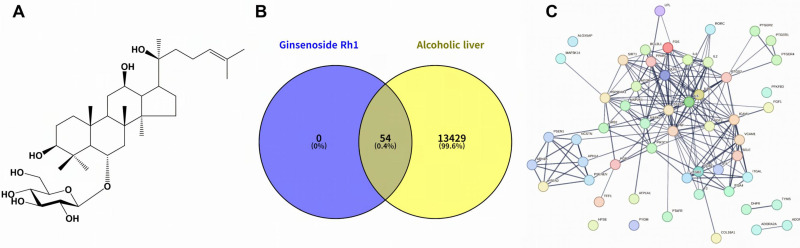

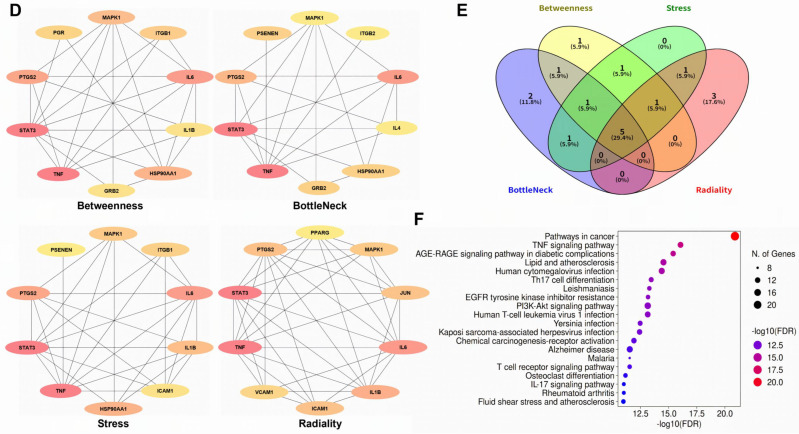

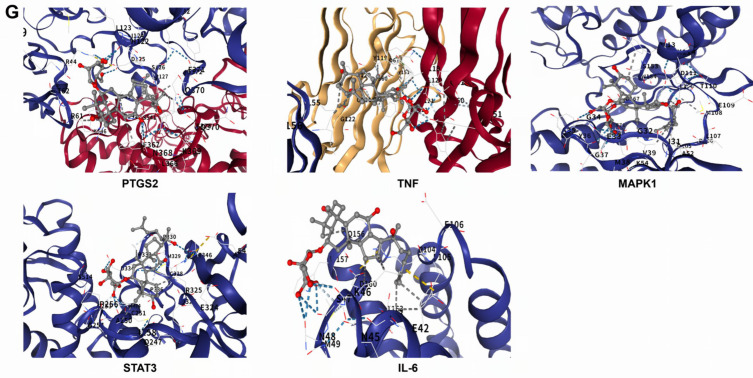
Identification of common targets, core hub genes, key pathways, and molecular docking analysis of ginsenoside Rh1 associated with alcoholic liver disease. (**A**) Chemical structure of Rh1, (**B**) Venn diagram showing common targets between Rh1 and alcoholic liver disease, (**C**) PPI network of common targets based using the STRING database with a confidence score > 0.7, and (**D**) identification of core hub genes based on network topological analysis. (**E**) Venn diagram of hub genes derived from network topological analysis. (**F**) KEGG pathway enrichment analysis of common targets, using a bubble chart. (**G**) Molecular docking analysis of Rh1 with core hub proteins.

Venn diagram analysis revealed 54 common targets between Rh1 (Figure 6A) associated targets and 13,483 genes linked to alcoholic liver disease (Figure 6B). The STRING-derived PPI network comprised 50 nodes and 195 edges, with an average node connectivity of 8.39, indicating dense protein interactions (Figure 6C). Key targets were identified by analyzing the top 10 nodes, ranked based on their betweenness, bottleneck, stress, and radiality scores (Figure 6D). TNF, PTGS2, MAPK1, STAT3, and IL-6 were identified as core hub genes (Figure 6E). IL-6 and TNF are inflammatory cytokines that activate the JAK–STAT3, NF-κB, and MAPK signaling pathways. MAPK1 and STAT3 regulate inflammatory responses, while PTGS2 functions as a COX-2 inducer. These core hub genes are established regulators of inflammatory pathways.

#### 3.8.2. Enriched GO Terms and KEGG Pathways of the Common Targets

GO enrichment analysis of the 54 common Rh1-alcoholic liver disease targets revealed consistent patterns across all categories. In the Biological Process category (Figure S1A), the responses to organonitrogen compounds, inflammation, and organic substances were prominent, suggesting that Rh1 regulates metabolic and inflammatory responses. In the Molecular Function category (Figure S1B), enriched GO terms included protein binding, signaling receptor binding, and growth factor receptor binding, suggesting that Rh1 may influence intracellular signaling through interactions with multiple receptors and growth factors. In the Cellular Component category (Figure S1C), the target proteins were mainly located in the gamma-secretase and plasma membrane protein complexes. KEGG pathway enrichment analysis (Figure 6F) revealed the TNF, PI3K–Akt, Th17 cell differentiation, and IL-17 signaling pathways as significantly enriched [31,32,33].

#### 3.8.3. Results of Molecular Docking

Docking simulations between ginsenoside Rh1 and the five core hub proteins showed binding affinities of −7.5 to −9.4 kcal/mol (Figure S2). Notably, PTGS2 and TNF exhibited the highest binding affinities, each at −9.4 kcal/mol. For PTGS2 (PDB ID: 5KIR), BIOVIA Discovery Studio analysis showed that Rh1 stabilized binding via hydrogen bonds with Phe367 (1.89 Å) in Chain A, and Arg44 (2.40 Å, 2.74 Å) and His122 (2.04 Å) in Chain B. An alkyl interaction with Pro542 (4.10 Å) in Chain A further contributed to hydrophobic stabilization. Van der Waals interactions with amino acid residues, including Ala543, Lys369, and Asn368, further stabilized Rh1 in the PTGS2 binding pocket (Figure 6G and Figure S2A). Docking analysis of TNF (PDB ID: 2AZ5) showed hydrogen bonds with Tyr151 (2.93 Å) in Chain A and Gly121 (2.95 Å, 3.18 Å) in Chain B. Hydrophobic interactions with Leu55 (5.12 Å) in Chain D and van der Waals interactions with several amino acid residues, including Gln61, Tyr59, Ser60, and additional surrounding residues, stabilized the ligand (Figure 6G and Figure S2B). MAPK1 (PDB ID: 1TVO) had the third-highest binding affinity at −9.1 kcal/mol. Rh1 formed hydrogen bonds with Asp111 (2.80 Å) and Gly34 (1.91 Å, 3.09 Å) in Chain A. Hydrophobic interactions occurred with Ala52 (4.10 Å), Val39 (4.25 Å), and Ile31 (5.27 Å). Van der Waals interactions with Met108, Thr110, Leu156, and Gly32 further stabilized the complex (Figure 6G and Figure S2C). STAT3 (PDB ID: 6NJS) had the fourth-highest binding affinity at −8.0 kcal/mol. Rh1 formed hydrogen bonds with Thr346 (2.71 Å), Cys251 (2.18 Å), Ala250 (2.69 Å), Asp334 (2.64 Å), and Arg325 (3.09 Å) in Chain A, while Pro333 (4.70 Å) participated in hydrophobic interactions. Van der Waals interactions with Pro330, Pro336, and Cys328 further stabilized the complex (Figure 6G and Figure S2D). IL-6 (PDB ID: 1ALU) had a lower binding affinity of −7.5 kcal/mol, still below −7.0 kcal/mol, indicating significant binding potential. Rh1 formed hydrogen bonds with Lys66 (1.91 Å, 2.76 Å) and Lys86 (3.31 Å, 2.97 Å) in Chain A, while hydrophobic interactions involved Leu165 (4.73 Å) and Arg168 (4.82 Å). Van der Waals interactions with Lys70, Ala68, Met67, and Asn61 further stabilized the complex (Figure 6G and Figure S2E).

The re-docking analysis for protocol validation yielded RMSD values ranging from 0.02 to 2.172 Å. While the RMSD for IL-6 (2.172 Å) slightly exceeded the conventional 2.0 Å threshold, all ligands were found to occupy positions in close proximity to their original binding sites (Figure S3).

## 4. Discussion

Alcohol-related liver disease resulting from persistent excessive alcohol intake remains a major global health concern. This study comprehensively evaluated the hepatoprotective effects of BGE and its major ginsenosides, using in vivo, in vitro, and in silico approaches. In vivo, the anti-inflammatory and hepatoprotective effects of BGE were confirmed in an acute EtOH-induced liver injury mouse model. Since alcoholic liver damage is dose- and time-dependent, an intragastric EtOH-feeding model that precisely controls alcohol intake is useful for studying its effects [35]. As the mouth is the main pathway of alcohol intake in humans, intragastric feeding provides a more reliable model for studying alcoholic liver pathophysiology. Sex- and strain-dependent differences are recognized as important factors in ALD. Female mice have been shown to exhibit greater susceptibility to ethanol-induced steatosis and inflammatory responses, particularly in short-term models, which is consistent with clinical observations of increased vulnerability in women, which is likely due to enhanced endotoxin sensitivity and inflammatory activation. Although C57BL/6 mice are widely used in ALD research, BALB/c mice also represent a well-characterized model for inflammation- and oxidative stress-related liver injury and have been successfully applied in ethanol-induced liver injury studies [36,37]. Therefore, female BALB/c mice were selected as a suitable and reproducible model to sensitively evaluate the inflammatory and therapeutic effects examined in this study. Early-stage alcoholic liver disease progresses from small to large fat droplets in hepatocytes, leading to fatty hepatitis and, eventually, liver cirrhosis from fibrosis [38]. In this study, EtOH induced hepatic fat accumulation, which was reduced via BGE treatment.

AST, ALT and LDH activities are low under normal conditions, but liver damage releases these enzymes into the bloodstream, raising their serum levels. Thus, measuring serum AST and ALT is a reliable tool for diagnosing liver damage [14]. AILD contributes to alcohol-induced hepatitis, primarily through oxidative stress from reduced antioxidant activity or excess free radicals [38]. CAT, the most abundant liver enzyme, catalyzes the decomposition of hydrogen peroxide into water and oxygen [39]. Alcohol-damaged livers show reduced CAT activity due to excessive intracellular radicals that overwhelm or inhibit the enzyme. SOD is a potent antioxidant that converts toxic superoxide anions into hydrogen peroxide [40]. The BGE treatment reduced serum AST and ALT in EtOH-fed mice and significantly increased CAT and SOD activities.

Alcohol-induced oxidative stress triggers immune and inflammatory responses in multiple liver cell types, causing liver injury [5,6]. NO, an inflammatory mediator, primarily helps to eliminate infectious agents; however, excessive and unregulated NO production induced by strong stimuli such as alcohol damages normal cells and triggers local or systemic inflammatory responses [41]. NO secreted from inflammatory cells, including macrophages, is generated through iNOS expression, which is stimulated by inflammatory cytokines such as TNF-α and IL-6 [42]. COX-2 is critical in the conversion of arachidonic acid to prostaglandins, which regulate immune functions [43]. Proinflammatory cytokines, including TNF-α, interleukin-1β, and IL-6, promote inflammation and are produced by stimulated macrophages [44]. HO-1 is an inducible, rate-limiting enzyme in heme catabolism that provides cellular protection in preclinical injury models by inhibiting apoptosis, inflammation, and cell proliferation [45,46]. The BGE treatment significantly suppressed inflammatory mediator expression (iNOS and COX-2) and proinflammatory cytokines (TNF-α and IL-6), while enhancing HO-1 expression in EtOH-induced HepG2 cells. To investigate the molecular mechanism underlying the protective effect of BGE against EtOH-induced inflammatory liver damage, this study focused on ginsenoside Rh1, which was present at relatively high levels alongside the major components Rg3, Rk1, and Rg5 to assess its role in the observed biological activity. Rh1 is a compound produced through the structural transformation of major saponins during ginseng heat treatment and steaming [34]. Previous studies report that Rh1 exhibits anti-inflammatory activity by suppressing iNOS and COX-2 expression through regulation of the NF-*κ*B pathway activation [47]. Consistent with these findings, Rh1 significantly suppressed the expression of inflammatory enzymes iNOS and COX-2 in EtOH-treated HepG2 cells and reduced the levels of the proinflammatory cytokines TNF-α and IL-6, key initiators of inflammatory responses. Simultaneously, Rh1 increased the expression of the cytoprotective factor HO-1, indicating a dual effect of suppressing inflammation and enhancing cellular protection. These experimental results may be associated with the findings of the in silico network pharmacology and molecular docking analyses. KEGG pathway enrichment analysis of the 54 common targets revealed the involvement of major inflammatory signaling pathways, including TNF, PI3K–Akt, Th17 cell differentiation, and IL-17 signaling [31,32,33]. Furthermore, topological analysis identified five core hub target genes—PTGS2, TNF, MAPK1, STAT3, and IL-6—supporting the possibility that Rh1 may exert multi-target regulatory effects on inflammatory pathways involved in alcoholic liver disease.

Repeated alcohol consumption causes acetaldehyde accumulation and excessive oxidative stress in the liver, which activates Kupffer cells and triggers the release of TNF-α and IL-6 [48,49]. During this process, oxidative stress enhances the phosphorylation of the MAPK1 (ERK) and p38 MAPK signaling pathways, thereby amplifying TNF-α production [50]. Elevated TNF-α induces hepatocyte apoptosis and stimulates iNOS expression, worsening nitrosative stress and promoting hepatic lipid accumulation (steatosis) [48,51]. Secreted IL-6 concurrently activates the JAK/STAT3 signaling pathway, promoting hepatocyte survival and regeneration as part of a protective response. Under inflammatory conditions, IL-6 signaling also activates the MAPK1 (ERK) pathway, driving cell proliferation and inflammatory signaling cascades [50,52]. Sustained activation of IL-6/STAT3 and TNF-α pathways induces overexpression of downstream inflammatory mediators, including PTGS2 (COX-2) and iNOS, creating a vicious cycle that amplifies inflammatory responses, lipid peroxidation, and progressive liver injury [51,53]. A crucial mechanism for preventing the progression to more severe inflammation is the blockade of inflammatory signal transmission from the extracellular stimuli to the nucleus [53]. In this study, the core hub target gene MAPK1 (ERK2) serves as a central mediator, transmitting inflammatory signals from the cytoplasm to nuclear transcription factors and amplifying the inflammatory responses [54]. The inhibitory effects of Rh1 on iNOS and COX-2 expression suggest that it may suppress the progression to more severe inflammation, possibly by blocking the nuclear translocation of inflammatory signals, potentially through regulation of MAPK1 activity, which functions as an upstream signaling transmitter. Furthermore, Rh1 may potentially contribute to the regulation of STAT3 signaling, a key pathway involved in cell survival during inflammatory responses [52,53]. Through these mechanisms, Rh1 may be associated with hepatocyte protection while potentially promoting apoptosis in excessively proliferating inflammatory cells, thereby contributing to inflammatory homeostasis.

Molecular docking simulations suggested potential interactions between Rh1 and several core hub proteins, including PTGS2, TNF, MAPK1, STAT3, and IL-6, with predicted binding energies ranging from −7.0 to −9.4 kcal/mol. To assess the reliability of these docking results, a re-docking validation was performed. Most targets yielded RMSD values below 2.0 Å, indicating the high reproducibility of the predicted binding poses.

Among the analyzed target proteins, PTGS2 showed a relatively high predicted binding affinity. Structural studies have shown that classical COX-2 inhibitors bind within the cyclooxygenase active site, involving residues such as Arg120, Tyr355, Tyr385, and Ser530 [55]. However, the predicted Rh1 binding pose did not overlap with this canonical inhibitor binding region, suggesting that Rh1 may bind to an alternative region of PTGS2, rather than the canonical cyclooxygenase inhibitor pocket.

TNF is a homotrimeric cytokine consisting of three identical subunits. Structural analysis of TNF in a complex with a small-molecule ligand revealed that the ligand binds within a shallow pocket located at the interface between TNF subunits. In this structure, the ligand interacts with residues including Tyr59, Ser60, Gln61, Tyr119, Leu120, Gly121, Gly122, and Tyr151, which are normally buried within the trimer interface. The binding of the small molecule destabilizes the trimeric assembly by displacing one subunit, resulting in the formation of a TNF dimer–ligand complex while preserving the overall fold of the remaining subunits [56]. In the present docking analysis, Rh1 was predicted to interact with several residues located within this reported interfacial region. Specifically, a conventional hydrogen bond interaction was observed with Tyr151, while Gly121, Tyr59, Ser60, Gln61, Tyr119, and Leu120 were involved in van der Waals interactions. These residues correspond closely to those forming the small-molecule binding pocket identified in the TNF crystal structure. Therefore, the present findings suggest that Rh1 may interact with the TNF interfacial region that was previously reported for small-molecule binding.

MAPK1 contains a catalytic cleft that accommodates the ATP-binding pocket, which serves as a major ligand binding region for small molecules. Structural studies have shown that ligand interactions within this pocket involve residues such as Met108, Lys54, Gln105, Asp106, Leu156, and Cys166 [57]. In the present docking analysis, Rh1 was predicted to interact with MAPK1 through hydrogen bonds with Asp111 and Gly34, along with hydrophobic and van der Waals contacts involving residues including Met108, Lys54, Gln105, and Leu156. Because several of these residues are located within or near the ATP-binding region, Rh1 may interact with residues located within or near the ATP-binding pocket of MAPK1.

STAT3 contains a ligand-binding site within the SH2 domain, as revealed by the co-crystal structure, where the ligand interacts with residues including Arg609, Ser611, Ser613, Tyr640, Gln644, and Lys658, and the hydrophobic pocket formed by Ile659, Met660, and Leu666 [58]. In contrast, the docking analysis suggested that Rh1 may interact with residues located around the 250–350 region, which is spatially distinct from the canonical SH2 binding pocket, indicating a potential alternative binding region for Rh1 under the present docking conditions.

However, compared with the other proteins, IL-6 showed a slightly higher RMSD value in the re-docking analysis. IL-6 adopts a four-helix bundle structure and utilizes three major receptor binding sites. Site 1 interacts with IL-6Rα, whereas Sites 2 and 3 form the binding interface for gp130. Although Sites 2 and 3 are relatively flat, small pockets are present that may provide potential regions that interfere with gp130 association [59]. In the present IL-6 re-docking analysis, an RMSD value of 2.172 Å was obtained. This value indicates substantial similarity to the native ligand coordinates in the crystal structure, although minor differences in the detailed binding pose were observed [60]. In the crystallographic structure, L(+)-tartaric acid binds to Site 1 through direct hydrogen bonds with Arg182, Arg179, and Gln175 in Helix D. The re-docked ligand was found to interact with adjacent residues within Helix D, including Ser176, Glu172, and Phe173, occupying a proximal position to the original binding residues. Although the RMSD slightly exceeded the conventional 2.0 Å success criterion, the overall binding orientation was maintained, suggesting that the deviation remains within an acceptable range for preserving the structural relevance of the protein–ligand interaction [60].

In the present docking analysis, Rh1 was predicted to interact with residues located in the loop region between helices A and B and near helix D, including Asn61, Leu62, Asn63, Leu64, Pro65, Lys66, Met67, Ala68, Lys70, Arg168, Ser169, and Glu172. According to the reported IL-6 crystal structure, residues Ala61–Glu69 form an extended loop that exposes hydrophobic side chains into a cleft between helices B and D [59]. This binding pose suggests that Rh1 may occupy the structural cleft region of IL-6. However, because IL-6 signaling is primarily mediated through protein–protein interactions with IL-6Rα and gp130, these docking results should be interpreted cautiously and may represent potential ligand–protein interactions, rather than direct inhibition of cytokine signaling.

Overall, the docking analysis suggests that Rh1 may interact with several proteins involved in inflammatory signaling pathways, including upstream inflammatory cytokines (TNF-α and IL-6) and downstream mediators such as MAPK1 and STAT3, which may ultimately be associated with the modulation of PTGS2 (COX-2) expression, a key downstream inflammatory effector. However, because cytokines such as TNF and IL-6 primarily function through protein–protein interactions, the docking results should be interpreted cautiously and require further experimental validation.

**Figure 7:**
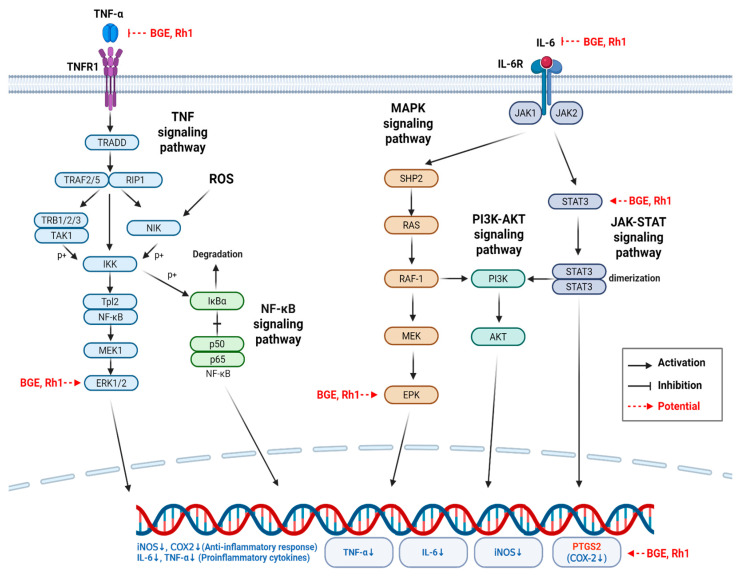
Overview of the proposed multi-target regulatory actions of BGE and Rh1 on key inflammatory mediators involved in alcohol-induced liver injury.

By integrating these results with the JAK/STAT3, MAPK, and PI3K/Akt signaling pathways based on the KEGG pathway database [31,32,33] and established mechanisms from the previous literature [52,53], the present work suggests the possibility that ginsenoside Rh1 may contribute to the coordinated, multi-target regulation of key inflammatory signaling pathways involved in alcoholic liver injury (Figure 7). While further experimental validation is required, the current in silico network pharmacology and molecular docking analyses provide mechanistic insights into how BGE-derived Rh1 may modulate inflammation throughout disease progression.

Collectively, these findings suggest that BGE, particularly Rh1, mitigates EtOH-induced liver damage by modulating oxidative stress and inflammatory signaling pathways, thereby preventing the transition to more severe inflammation and demonstrating its potential as a multi-target therapeutic candidate for alcoholic liver disease.

## Data Availability

The original contributions presented in this study are included in the article and Supplementary Materials. Further inquiries can be directed to the corresponding author.

## Supplementary Materials

The following supporting information can be downloaded at: https://www.mdpi.com/article/10.3390/antiox15040461/s1.

## Abbreviations

The following abbreviations are used in this manuscript:

AILD	Alcohol-induced liver damage
ALT	Alanine aminotransferase
AST	Aspartate aminotransferase
BGE	Black ginseng extract
CAT	Catalase
COX-2	Cyclooxygenase-2
CYP2E1	Cytochrome P450 2E1
EGR1	Early Growth Response 1
EtOH	Ethanol
−ESI	Negative electrospray ionization mode
FBS	Fetal bovine serum
GO	Gene ontology
H&E	Hematoxylin and eosin
HDE	*Hovenia dulcis* extract
HO-1	Heme oxygenase-1
IL-6	Interleukin-6
iNOS	Inducible nitric oxide synthase
JAK2	Janus kinase 2
KEGG	Kyoto Encyclopedia of Genes and Genomes
LDH	Lactate dehydrogenase
MAPK1	Mitogen-activated protein kinase 1
MRM	Multiple reaction monitoring
NO	Nitric oxide
PCR	Polymerase chain reaction
PPI	Protein–protein interaction
PTGS2	Prostaglandin-endoperoxide synthase 2
RMSD	Root mean square deviation
ROS	Reactive oxygen species
SD	Standard deviation
SOD	Superoxide dismutase
STAT3	Signal transducer and activator of transcription 3
TNF	Tumor necrosis factor
UPLC	Ultra-performance liquid chromatography
UPLC–Q-TOF/MS	Quadrupole time-of-flight mass spectrometry coupled with UPLC
UPLC–Q-TRAP–MS/MS	Quadrupole linear ion trap tandem mass spectrometry coupled with UPLC
