# Supplementary material for: Hepatoprotective Effects of Black Ginseng Extract and Ginsenoside Rh1 Against Alcohol-Induced Liver Injury: Mechanistic Insights from Network Pharmacology, In Vitro, and In Vivo Analysis
Source: Antioxidants (Basel). 2026 Apr 8;15(4):461. doi: 10.3390/antiox15040461 (PMC13113838; doi:10.3390/antiox15040461)
Supplement: Supplementary file 1 [file antioxidants-15-00461-s001.zip › antioxidants-4186613-supplementary.pdf]

Supplementary Information

Hepatoprotective Effects of Black Ginseng Extract and Ginsenoside Rh1 Against Alcohol-Induced Liver Injury: Mechanistic Insights from Network Pharmacology, In Vitro, and In Vivo Analysis

Hyeon Seon Na et al.

● Supplementary Figures and Tables

Figure S1. Bubble chart of GO enrichment analysis of common targets. (A) Biological process (BP), (B) molecular function (MF), and (C) cellular component (CC).

Figure S2. Molecular docking analysis of Ginsenoside Rh1 with core hub proteins. (A) PTGS2, (B) TNF, (C) MAPK1, (D) STAT3, (E) IL-6 and (F) summary of molecular docking results.

Figure S3. Redocking validation of the docking protocol for representative target proteins. (A) 5KIR, (B) 2AZ5, (C) 1TVO, (D) 6NJS, and (E) 1ALU. For each target, (1) superposition of crystallographic and re-docked ligand poses (gray, crystallographic; red, re-docked), (2) crystallographic ligand–protein interactions visualized using BIOVIA Discovery Studio, and (3) re-docked ligand–protein interactions. (F) Summary of redocking metrics for target proteins.

Table S1. Analytical conditions and results for the determination of ginsenoside Rh1, using UPLC-Q-TRAP-MS/MS.

Table S2. Summary of metabolite profiling data of the black ginseng extract obtained by UPLC-Q-TOF/MS.

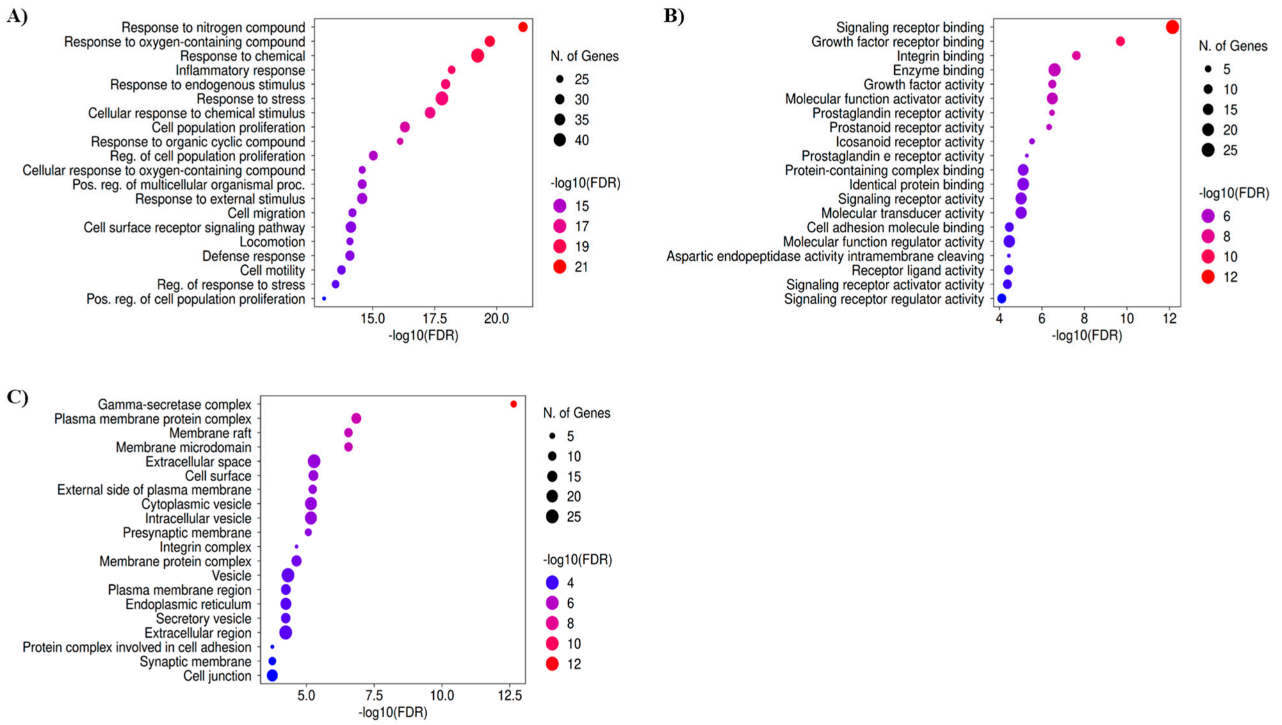

Figure S1. Bubble chart of GO enrichment analysis of common targets. (A) Biological process (BP), (B) molecular function (MF), and (C) cellular component (CC).

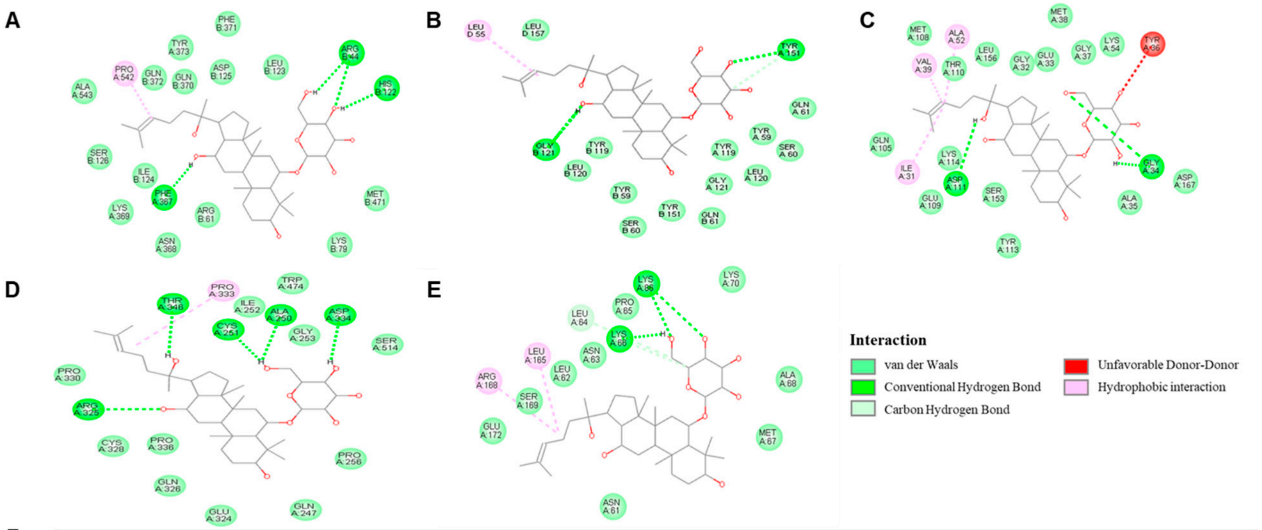

F

Target	Vina score	Conventional hydrogen bond (Å)	Hydrophobic interaction (Å)	Carbon hydrogen bond (Å)	Van der Waals
**PTGS2**	-9.4	A : Phe367 (1.89), B : Arg44 (2.4, 2.74), His122 (2.04)	A : Pro542(4.1)		A : Ala543, Lys369, Asn368, B : Phe371, Leu123, Asp125, Tyr373, Gln370, Gln372, Ser126, Ile124, Arg61, lys79, Met471
**TNF**	-9.4	A : Tyr151(2.93), B : Gly121(2.95, 3.18)	D : Leu55(5.12)	A : Tyr151(3.18)	A : Gln61, Tyr59, Ser60, Tyr119. Leu120, Gly121, B : Gln61, Tyr151, Ser60, Tyr59, Tyr119, Leu120, D : Leu157
**MAPK1**	-9.1	A : Asp111(2.80), Gly34(1.91, 3.09)	A : Ala52(4.10), Val39(4.25), Ile31(5.27)		A : Met108, Thr110, Leu156, Gly32, Glu33, Gly37, Met38, Lys54, Asp167, Ala35, Ser153, Glu109, Lys114, Gln105
**STAT3**	-8.0	A : Thr346(2.71), Cys251(2.18), Ala250(2.69), Asp334(2.64), Arg325 (3.09)	A : Pro333(4.70)		A : Pro330, Pro336, Cys328, Gln326, Glu324, Gln247, Pro256, Ser514, Gly253, Ile252, Trp474
**IL6**	-7.5	A : Lys66(1.91, 2.76), Lys86 (3.31, 2.97)	A : Leu165(4.73), Arg168(4.82)	A : Leu64(3.65)	A : Lys70, Ala68, Met67, Asn61, Glu172, Ser169, Leu62, Asn63, Pro65

Figure S2. Molecular docking analysis of Ginsenoside Rh1 with core hub proteins. (A) PTGS2, (B) TNF, (C) MAPK1, (D) STAT3, (E) IL-6 and (F) summary of molecular docking results.

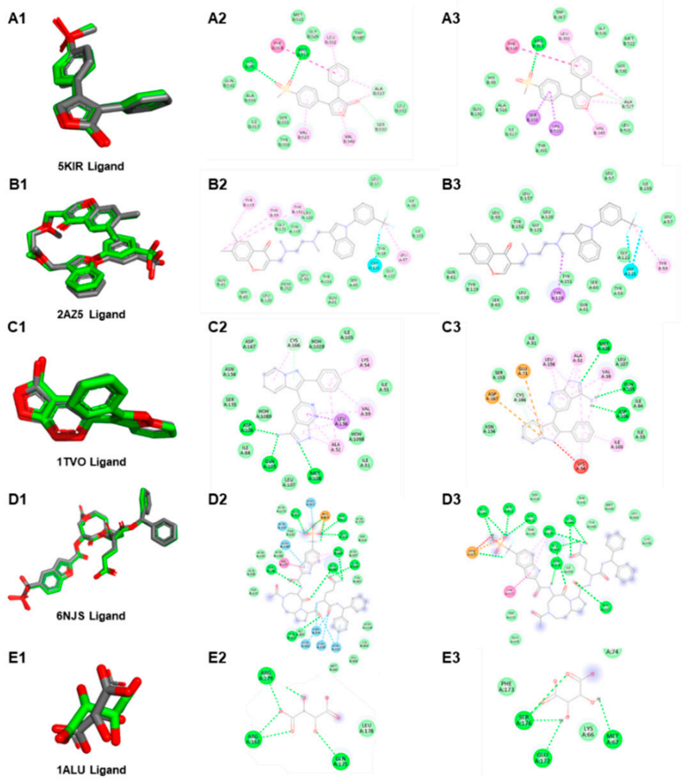

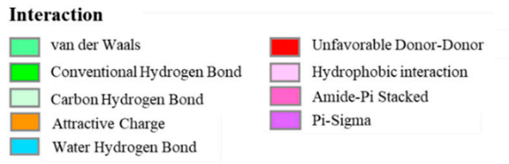

F

Target	PDB ID	Ligand	Vina score	RMSD
PTGS2	5KIR	Rofecoxib	-10.2	0.02
TNF	2AZ5	6,7-dimethyl-3-[(methyl{2-[methyl({1-[3-(trifluoromethyl)phenyl]-1H-indol-3-yl}methyl)amino]ethyl}amino)methyl]-4H-chromen-4-one	-9.2	0.648
MAPK1	1TVO	5-(2-phenylpyrazolo[1,5-a]pyridin-3-yl)-1H-pyrazolo[3,4-c]pyridazin-3-amine	-10.1	1.163
STAT3	6NJS	[(2-{[(5S,8S,10aR)-3-acetyl-8-({(2S)-5-amino-1-[(diphenylmethyl)amino]-1,5-dioxopentan-2-yl}carbamoyl)-6-oxodecahydropyrrolo[1,2-a][1,5]diazocin-5-yl]carbamoyl} -1H-indol-5-yl)(difluoro)methyl]phosphonic acid (non-preferred name)	-8.4	0.852
IL6	1ALU	L(+)-tartaric acid	-3.7	2.172

Figure S3. Redocking validation of the docking protocol for representative target proteins. (A) 5KIR, (B) 2AZ5, (C) 1TVO, (D) 6NJS, and (E) 1ALU. For each target, (1) superposition of crystallographic and re-docked ligand poses (gray, crystallographic; red, re-docked), (2) crystallographic ligand–protein interactions visualized using BIOVIA Discovery Studio, and (3) re-docked ligand–protein interactions. (F) Summary of redocking metrics for target proteins.

Table S1. Analytical conditions and results for the determination of ginsenoside Rh1 using UPLC-Q-TRAP-MS/MS.

A) Parameters of ginsenoside Rh1 to MRM mode in UPLC-Q-TRAP-MS/MS.

Compound	Q1	Q3	Time	DP	EP	CEP	CE	CXP
Ginsenoside Rh1	637.274	160.9	150	-70	-8.5	-28	-28	0

B) Identification of ginsenoside Rh1 by UPLC-Q-TRAP-MS/MS.

Compound	Molecular Formula	Molecular Weight	Measured Value $[M\text{-}H]^-$	MS/MS Fragmentations
Ginsenoside Rh1	$C_{36}H_{62}O_9$	638.87	637.2	637.2[M-H]-; 553.2[M-C6H11-H]-; 475.2[M-Glu-H]-; 457.4[M-Glu-H2O-H]-; 391.2[M-Glu-C6H12-H]-; 373.2[M-Glu-C6H11-H2O-H]-; 347.2[M-Glu-C8H15O-H]-; 328.8[M-Glu-C8H17O2-H]-; 178.6[M-Aglycone-H]-; 161.0[M-Aglycone-H2O-H]-; 143.2[M-Glu-C22H35O2-H]-; 113.0[M-Glu-C24H39O3-H]-; 84.8[M-Glu-C24H40O4-H]-;

C) Quantification of ginsenoside Rh1 in black ginseng extract.

Compound	Calibration Curves	Correlation Coefficient	Linear Range (ppm)	Content (mg/g)	LOD	LOQ
Ginosenoside Rh1	Y = 0.813x + -64.9	0.9998	0.3125 ~ 10	4.73	0.429	1.431

Table S2. Summary of metabolite profiling data of the black ginseng extract obtained by UPLC-Q-TOF/MS.

NO.	Ginsenoside	Observed RT (min)	Observed m/z	Observed m/z	Mass error (ppm)[a]	Area	Relative percentages (%)
1	notoginsenoside R1	4.09	977.5303	977.5303	-4	1094	0.008
2	vinaginsenoside R4	11.28	1007.541	1007.541	-1.8	1162.333	0.009
3	ginsenoside Rf	12.2	845.489	845.489	-0.7	952254.3	7.190
4	notoginsenoside R2	12.92	815.4792	815.4792	-0.8	563597.3	4.256
5	notoginsenoside R4	13.38	1285.644	1285.644	-0.7	5104.333	0.039
**6**	**ginsenoside Rh1**	**13.64**	**683.4385**	**683.4385**	**0.3**	**1435233**	**10.837**
7	ginsenoside F5	13.67	815.4793	815.4793	-1	143208	1.081
8	ginsenoside Rg2	13.79	829.4959	829.4959	0.4	1015503	7.668
9	20(R)-ginsenoside Rg2	14.11	829.4955	829.4955	-0.9	580368	4.382
10	20(R)-ginsenoside Rh1	14.12	683.438	683.438	-0.1	1099959	8.306
11	ginsenoside F3	14.21	815.4789	815.4789	-0.7	1369	0.010
12	ginsenoside Rs3	18.85	871.5041	871.5041	-2.4	2749.667	0.021
13	gypenoside XVII	20.92	991.5452	991.5452	-2	2363.667	0.018
14	gypenoside L	25.36	845.4811	845.4811	-3.3	1290.333	0.010
15	notoginsenoside Ft1	25.75	961.5376	961.5376	-1.1	6222	0.047
16	Protopanaxatiol	25.9	521.3845	521.3845	-0.4	850	0.006
17	ginsenoside Rg3	26.17	829.4936	829.4936	1.1	2460214	18.576
18	ginsenoside Mc	26.54	799.4832	799.4832	0	4765.333	0.036
19	compound Y	26.7	799.4836	799.4836	-2.8	18254	0.138
20	ginsenoside Rk1	27.6	811.4844	811.4844	-1.1	2468830	18.641
21	ginsenoside Rg5	27.73	811.4853	811.4853	-2.3	2296054	17.337
22	ginsenoside Rh2	27.86	667.4419	667.4419	-2.6	74218.33	0.560
23	ginsenoside Rk2	29.16	649.4315	649.4315	-2.5	49552.67	0.374
24	ginsenoside Rh3	29.28	649.4315	649.4315	-1.6	59522.33	0.449

[a] Mass accuracy < 5 ppm.
